# Damage Identification Using Measured and Simulated Guided Wave Damage Interaction Coefficients Predicted Ad Hoc by Deep Neural Networks

**DOI:** 10.3390/s25061681

**Published:** 2025-03-08

**Authors:** Christoph Humer, Simon Höll, Martin Schagerl

**Affiliations:** 1 Institute of Structural Lightweight Design, Johannes Kepler University Linz, Altenbergerstr. 69, 4040 Linz, Austria; martin.schagerl@jku.at; 2 Independent Researcher, Am Fuchsholz 15, 07381 Wernburg, Germany

**Keywords:** guided waves, structural health monitoring, damage identification, wave damage interaction coefficients, deep neural networks, machine learning, principal component analysis

## Abstract

Thin-walled structures are widely used in aeronautical and aerospace engineering due to their light weight and high structural performance. Ensuring their integrity is crucial for safety and reliability, which is why structural health monitoring (SHM) methods, such as guided wave-based techniques, have been developed to detect and characterize damage in such components. This study presents a novel damage identification procedure for guided wave-based SHM using deep neural networks (DNNs) trained with experimental data. This technique employs the so-called wave damage interaction coefficients (WDICs) as highly sensitive damage features that describe the unique scattering pattern around possible damage. The DNNs learn intricate relationships between damage characteristics, e.g., size or orientation, and corresponding WDIC patterns from only a limited number of damage cases. An experimental training data set is used, where the WDICs of a selected damage type are extracted from measurements using a scanning laser Doppler vibrometer. Surface-bonded artificial damages are selected herein for demonstration purposes. It is demonstrated that smart DNN interpolations can replicate WDIC patterns even when trained on noisy measurement data, and their generalization capabilities allow for precise predictions for damages with arbitrary properties within the range of trained damage characteristics. These WDIC predictions are readily available, i.e., ad hoc, and can be compared to measurement data from an unknown damage for damage characterization. Furthermore, the fully trained DNN allows for predicting WDICs specifically for the sensing angles requested during inspection. Additionally, an anglewise principal component analysis is proposed to efficiently reduce the feature dimensionality on average by more than 90% while accounting for the angular dependencies of the WDICs. The proposed damage identification methodology is investigated under challenging conditions using experimental data from only three sensors of a damage case not contained in the training data sets. Detailed statistical analyses indicate excellent performance and high recognition accuracy for this experimental data-based approach. This study also analyzes differences between simulated and experimental WDIC patterns. Therefore, an existing DNN trained on simulated data is also employed. The differences between the simulations and experiments affect the identification performance, and the resulting limitations of the simulation-based approach are clearly explained. This highlights the potential of the proposed experimental data-based DNN methodology for practical applications of guided wave-based SHM.

## 1. Introduction

Thin-walled structures occur in many aeronautical and aerospace engineering applications, such as fuselages of aircraft or hulls of spaceships, where demands on the structures’ weight are paramount [1]. In these applications, thin-walled components not only entail constructive functionalities but also contribute to the overall structural performance. Thus, their integrity is of key importance for the overall structural safety and reliability. In addition to conventional inspections using non-destructive testing methods, structural health monitoring (SHM) can continuously provide information about the health state of critical structural components. Therefore, sensors and possibly actuators are permanently attached on or even integrated into a structure [2]. The resulting self-sensing functionality of these so-called smart structures enables advanced SHM procedures to complement conventional non-destructive evaluation methods for even safer and more reliable operation of structures [3]. Besides that, certain traditional non-destructive testing methods, e.g., optical crack detection [4], have the potential for automation, which can significantly increase accuracy while saving inspection time.

SHM methods typically exploit various physical phenomena that are altered by structural damage. For example, traditional strain-based approaches monitor strain distributions in critical areas, which change notably as damage occurs [5]. These variations can be detected using simple strain gauges [6] or fiber optical sensors [7]. Other sensor technologies are based on magnetic fields [8,9] or pneumatic principles [10]. Lightweight and inexpensive piezoelectric transducers, i.e., piezoelectric wafer active sensors (PWASs), are commonly employed in SHM applications and can be easily mounted to or seamlessly integrated into structures. These PWASs leverage the reversible piezoelectric effect, allowing for their dual function as both actuators and sensors, while even a simultaneous use of both functions is possible [11]. The alternative use by alternating their role provides exceptional versatility and makes them capable of employing several SHM methods, such as passive acoustic emission methods [12] or active guided wave-based [13] and electro-mechanical impedance techniques [14].

In particular, guided wave-based SHM methods have attracted significant attention in recent decades [15]. In a traditional pitch–catch configuration, possible damage influences the propagating guided waves on the way from the actuator to the sensor [16]. Hence, the sensor signal differs in comparison to a baseline from the assumed pristine condition [17]. Analyzing these differences allows us to draw certain conclusions about the damage, where the existence of damage is commonly revealed by baseline subtraction [18] or anomaly detection [19]. By using a typical sparse sensor network, e.g., an array of PWASs, the location of the damage can be determined by various imaging methods, e.g., delay-and-sum [20] or time reversal methods [21]. Advanced data processing algorithms even allow us to localize multiple damages based on coefficient matrices constructed from guided wave reflections [22]. Furthermore, tomographic imaging techniques can reconstruct the damage location, but they require a relatively dense sensor array [23,24]. This might hinder their application on large-scale structures.

Generally, SHM systems need smart and powerful computational techniques to achieve accurate results. Hence, state-of-the art SHM approaches increasingly combine traditional methods, e.g., guided wave-based techniques, with machine learning methods due to their high potential for efficient dissemination of information and knowledge discovery. For example, these machine learning approaches can create a model based on training data to predict or decide on a specific task without explicitly implementing the solution. The performance of such self-adaptive models improves solely by learning from the given training data. Artificial neural networks are inspired by biological neural networks and employ non-linear regression models, where the chain rule of differentiation and the gradient descent method are used to learn their parameters incrementally. Sbarufatti et al. [25] employed such an artificial neural network to locate and quantify fatigue cracks in aluminum plates using numerically simulated Lamb wave signals. Support vector machines use statistical learning theory to maximize the margins of selected data samples, i.e., support vectors. This optimization problem is typically solved in a recursive manner by dynamic programming to reveal optimal decision bounds. In the context of guided wave-based SHM, these support vector machines are utilized by Zhang et al. [26] to detect damages in an aluminum beam using numerical simulation data. Xu [27] proposed an impact identification system based on least squares support vector machines to estimate the location and amplitude of an impact. The classification and regression tasks in a guided wave-based SHM configuration were studied by Miorelli et al. [28] for automatic detection, localization and characterization of damages using non-linear support vector machines and a kernel-based feature mapping. Hoell and Omenzetter [29] present a damage localization technique based on hierarchical neuro-fuzzy models using experimental data from a laboratory-scale wind turbine blade. The proposed model architecture enables the application of neural network-related optimization techniques.

However, easily available high-computational-power and dedicated open-source software packages, e.g., TensorFlow [30], have empowered modern SHM methods based on highly complex models using so-called deep learning [31,32]. These deep learning algorithms typically utilize artificial neural networks with multiple layers to learn complex relationships between defined inputs and outputs by non-linear mappings, where several classes can be distinguished on the basis of different architectures or applications. Convolutional neural networks utilize two specific types of layers, i.e., convolutional layers and pooling layers, for their hidden layers in between input and output layers. The convolutional layer performs convolutions for feature extraction, and the pooling layer is used to compress the feature dimensionality. The subsequent fully connected layers derive the actual structural state from the given data by using non-linear mappings. Sony et al. [33] found that these convolutional neural networks are mainly utilized for time series analysis and vision-based approaches in SHM-related applications. Lomazzi et al. [34] proposed a framework for damage diagnosis using two convolutional neural networks, one for classification and one for regression, to determine the existence and location of a damaged area, respectively. An efficient learning architecture based on convolutional neural networks is presented by Ye and Toyama [35] to detect damages by analyzing ultrasonic wave propagation videos. The evaluation of several successive frames allows us to combine spatial and temporal information of the wavefield. Keshmiri Esfandabadi et al. [36] employed convolutional neural networks with compressive sensing to recover high spatial frequency information from low-resolution wavefields measured by a scanning laser Doppler vibrometer (SLDV). Gonzalez-Jimenez et al. [37] use these networks to accurately localize damages with different sizes in anisotropic composite plates. A multi-level classification technique using convolutional neural networks is proposed by Shao et al. [38], where the damage presence, location and size are the outputs of the different levels.

Another class of deep learning models is that of deep autoencoders. In SHM, they are typically used to reduce the feature dimensionality, where the input data are reproduced in the outputs by using hidden layers with lower dimensions. Abbassi et al. [39] compared their performance for compressing features to other different unsupervised learning techniques, e.g., principal component analysis (PCA), and presented promising results for damage detection and localization using a guided wave-based approach under varying temperature conditions. Lee et al. [40] proposed an automated diagnosis technique based on autoencoders to detect and characterize fatigue damages in composite materials. Gao and Hua [41] employ a hybrid approach that combines convolutional neural networks with a stacked autoencoder to analyze broadband guided wave signals for corrosion mapping. A two-step SHM approach for the classification of frequency domain features is presented by Mousavi et al. [42], where a deep neural network (DNN) is trained by a combination of an autoencoder and backpropagation algorithms. In general, these DNNs use multiple hidden layers to learn complex non-linear relationships between given input data and defined outputs. Bao et al. [43] employed them to detect anomalies due to potential damages in recorded acceleration data from a real SHM system monitoring a long-span bridge. They also discussed the influence of an unbalanced training data set on the recognition accuracy. A DNN-based SHM technique is presented by Yoon et al. [44] to detect and localize cracks by predicting the strain distribution from local strain measurements using a sparse array of strain gauges. The dimensions of the DNN predictions are efficiently reduced by employing PCA.

However, to obtain further information beyond the existence and location of a damaged area, advanced data processing of the guided wave signals is required. Balasubramaniam et al. [45] propose a hybrid global–local approach to efficiently quantify damages in composites using a pixel-based confusion matrix. Migot et al. [46] successfully estimate the size and orientation of a crack in an aluminum plate from experimental measurements using advanced imaging methods. In a different approach, the guided wave scattering, i.e., the interaction between the incident probing waves and the damage, is analyzed. Wu et al. [47] proposed a Bayesian framework for damage identification using a semi-analytical approach to model the wave damage interaction. The presented numerical and experimental scattering coefficients of a partly through-thickness hole in an aluminum plate show good agreement. The so-called wave damage interaction coefficients (WDICs) consider not only the amplitude but also the phase of the scattered wavefield around a damaged area to describe the scattering comprehensively [48]. Both amplitude and phase coefficients form characteristic scattering patterns and reveal sensible frequencies as well as sensing directions as shown by Humer et al. [49] in a detailed comparison of experimental and numerical WDICs. These WDICs uniquely describe the damage with its characteristics for the given host structure. Hence, Humer et al. [50] employed them as highly sensitive features for damage identification in a study using solely numerical simulation data. They propose the use of a DNN to learn the complex relationships between damage characteristics, e.g., size or orientation, and the corresponding WDICs from only a limited number of selected damage cases as training data. The smart interpolations of the trained DNN can predict WDICs even for unseen damage scenarios, which allows for creating a large WDIC database for damage identification. The calculation of such a large WDIC database by solely using numerical finite element (FE) simulations is computationally prohibitive.

In general, such simulation-based approaches have certain disadvantages that limit their applicability in practical SHM systems. Inherent modeling inaccuracies caused by inevitable model simplifications or uncertainties in the estimated numerical model parameters affect the accuracy of the model output. Hence, the development and validation of applicable numerical models can be challenging. These limitations do not apply to data-based techniques employing a statistical model solely built on experimental data. Therefore, this paper presents a novel damage identification procedure based on ad hoc WDIC predictions using experiment-based DNNs. This study proposes the use of DNNs to learn complex relationships between damage properties and imperfect experimental WDIC patterns measured by an SLDV. DNNs are chosen for their proven ability to model such highly non-linear relationships and their superior performance compared to simpler regression-based methods, as demonstrated in a prior study [50]. Additionally, the strong generalization capabilities of these DNNs enable accurate WDIC predictions, even when trained on a compact training data set of a few damage cases. These WDIC predictions are available within a relatively short time, i.e., ad hoc, and can be calculated directly on site during inspection. This innovative approach eliminates the need for a potentially storage-intensive database by utilizing customized WDIC predictions tailored for each specific scenario. This means, that for guided wave-based SHM applications using a sparse sensor array, the exact requested sensor positions can be employed to predict the WDICs as a reliable foundation for precise damage identification. The geometrical scaling property of WDICs enables the reuse of the already-trained DNNs for different but geometrically similar combinations of damage and host structure without any additional effort. Additionally, an anglewise PCA is proposed that not only compresses the high-dimensional WDIC features but also preserves their angular dependencies critical for accurate damage identification. Tailoring the dimensionality reduction to individual sensing angles enables optimal feature compression with improved accuracy and robustness. The proposed damage identification method is further tested in a simulation-based SHM approach. Here, another DNN is trained by numerical simulation data, and its predictions are employed to identify unknown damages from experimental measurement data in the same ad hoc manner. The performance of both data-based and simulation-based approaches is tested and statistically analyzed for a common three-sensor configuration by using 10,000 unique combinations of three different sensing angles. Furthermore, this study discusses an efficient technique for substantially reducing the dimension of the feature vector. This feature compression is based on an anglewise PCA conducted ad hoc individually for each sensing angle. Hence, the proposed novel technique significantly extends previous research and could pave the way towards future practical SHM applications.

The structure of this paper is as follows. First, the general methodology and the underlying theory of the WDICs, the DNN, the PCA and the damage identification procedure are explained. Second, details about the physical experiments, the numerical simulations, the DNN modeling and the anglewise PCA are described. Third, the damage identification results for different scenarios are presented and discussed. Fourth, the conclusion and future work are summarized.

## 2. Methodology and Theory

This paper proposes a damage identification method based on ad hoc WDIC predictions using DNNs. These WDICs uniquely describe the guided wave scattering due to damage and depend solely on the damage characteristics, i.e., type, size, orientation, etc., for a given host structure. This study uses reversible surface-bonded structural disturbances, i.e., artificial damages, as commonly employed in laboratory experiments using a pseudo-damage approach [37,38,51,52]. It is noteworthy that the presented methodology for damage identification is universally applicable and not limited to any specific type of damage. The artificial damages selected herein are solely for demonstration purposes and proof of concept. However, these artificial damages have square shapes with the properties thickness *t*, size *D* and orientation α with respect to the incident wave direction. The relations between these damage properties and the plate thickness *h* of the host structure define the dimensionless damage-to-plate thickness ratio $\tau =\frac{t}{h}$, the dimensionless damage size-to-thickness ratio $\delta =\frac{D}{t}$ and the dimensionless damage size-to-wavelength ratio $\Lambda =\frac{D}{\lambda }$. The latter uses the wavelength λ, which depends on the frequency *f* for a given plate thickness *h*. Using the characteristic product of frequency *f* and half plate thickness $d=\frac{h}{2}$ ensures identical dimensionless size-to-wavelength ratios Λ [49]. This allows for comparing the WDICs of geometrically similar damages directly.

**Figure 1:**
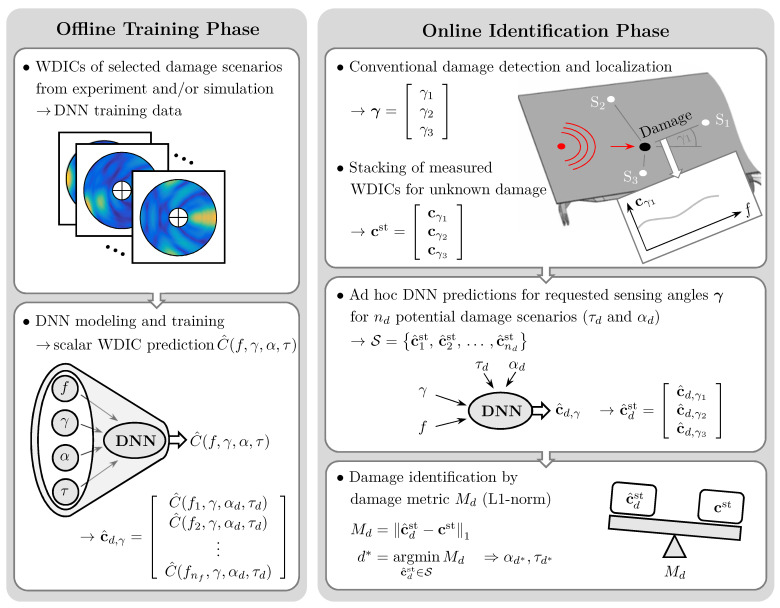
Damage identification methodology using ad hoc WDIC predictions by DNN.

The proposed damage identification method can be divided into two main parts, namely, an offline training and an online identification phase; see Figure 1. In the offline training phase, the WDICs of only a limited number of selected damage scenarios are used as training data set for a DNN. In this study, these WDICs are either experimentally measured using an SLDV or numerically simulated using an FE model. The orientation α and the dimensionless thickness ratio τ of the utilized artificial damages are varied to generate twelve different damage scenarios; see Table 1. The training data sets are used separately to train two different DNNs, i.e., an experiment-based and a simulation-based DNN, respectively. The generalization capabilities of these DNNs enable smart interpolations to predict WDICs with different frequency resolutions and arbitrary damage properties. Hence, the WDIC databases used for damage identification can be substantially extended [50].

In the online identification phase, a typical guided wave-based SHM setup is assumed [20,53]. Here, a structure is equipped with a sparse sensor array, e.g., PWASs. If damage occurs, it can be detected and localized by conventional methods, such as baseline subtraction [18] and time-of-flight evaluation [54], using one actuator and a minimum of three sensors. In practical applications, varying operational conditions, e.g., temperature or loading, can significantly influence guided wave propagation. Hence, the baseline subtraction must incorporate such variations to effectively isolate the scattered waves originating from the damage without artifacts in the measurement signals [55,56]. However, in this study, the existence and position of the damage are assumed to be known. This enables us to determine the sensing angle $\gamma_{i}$ in relation to the direction of the incident wave and the distance from the damage to the *i*-th sensor, respectively. The latter can be used to compensate for the different attenuation due to the geometrical spreading for each sensor prior to the identification [57]. By assuming a three-sensor setup, the damage feature vector of an unknown damage $c^{\mathrm{st}}={\left[\begin{array}{ccc} c_{\gamma_{1}}^{T} & c_{\gamma_{2}}^{T} & c_{\gamma_{3}}^{T} \end{array}\right]}^{T}$ is built by stacking the WDIC vectors $c_{\gamma_{i}}$ estimated from the three sensors ($S_{1}$, $S_{2}$ and $S_{3}$) according to their previously determined sensing angles $\gamma_{i}$.

The already-trained DNNs enable WDIC predictions of exactly these sensing angles $\gamma_{i}$ for many potential damage scenarios. The properties of these scenarios can be selected with respect to the training data set as well as practical considerations, such as required resolution or likeliness of occurrence. The calculation of the $n_{d}$ predictions only needs a very short time using DNNs. Thus, the prediction set S can be obtained ad hoc once the damage is detected and localized. Finally, the damage metric $M_{d}$ evaluates the damage feature vectors of the present unknown damage $c^{\mathrm{st}}$ and all predictions ${\hat{c}}_{d}^{\mathrm{st}}$ in the set $S=\left\{\begin{array}{c} {\hat{c}}_{1}^{\mathrm{st}},\;{\hat{c}}_{2}^{\mathrm{st}},\;\ldots \;,{\hat{c}}_{n_{d}}^{\mathrm{st}} \end{array}\right\}$ using the L1-norm. The minimal value of this damage metric indicates the identified damage $d^{*}$ with its properties.

This study uses experimental measurement and numerical simulation data for the training of two different DNNs. However, the proposed damage identification method was tested by experimental WDICs of a damage scenario unseen during training for both cases. Hence, the prediction accuracy of the DNN interpolations as well as the overall identification performance can be analyzed for a data-driven experimental DNN and a simulation-based model. Additionally, the length of the utilized feature vector can be significantly reduced by using the scores of an anglewise PCA. The damage identification results using these reduced feature vectors are presented in comparison, which results in a total of four different test cases, i.e., experiment-based and simulation-based DNN predictions using full and compressed feature vectors.

In the following, the theory of the WDICs as well as the DNN are explained in detail. Furthermore, the proposed damage identification method and the optional feature vector compression based on anglewise PCA are described.

### 2.1. Wave Damage Interaction Coefficients

The interaction of a probing guided wave packet and possible damage is generally complex, and analytical solutions are only available for geometrically well-defined cases, e.g., thickness changes [58] or through-thickness and partly through-thickness holes [59]. The combined analytical finite element method approach (CAFA) simulates the guided wave scattering using a detailed FE model and, thus, enables investigations of arbitrary damage geometries. Generally, the wave damage interaction generates a unique scattering pattern due to constructive and deconstructive interference in the scattered wavefield around the damage. The CAFA describes these scattering patterns by complex-valued WDICs, i.e., the amplitude and phase coefficients, and approximates the damage as a direction-dependent wave source [48]. The underlying idea is that the amplitude and phase of the incident wave are modified by these WDICs to result in the scattered wavefield. Further detailed information can be found in references [48,49,60]. However, the scalar amplitude coefficient $C_{AB}$ is defined by

(1) $$\begin{array}{c} C_{AB}\left(f,\gamma \right)=\left|\frac{u_{B}^{\mathrm{SC}}\left(f,\gamma \right)}{u_{A}^{\mathrm{IN}}\left(f\right)}\frac{1}{H_{1}^{\left(1\right)}\left(\xi_{B}\left(f\right)r_{\mathrm{sen}}\right)}\right| \end{array}$$

where $u_{A}^{\mathrm{IN}}\left(f\right)$ describes the out-of-plane displacement of the incident wavefield at the damage center position depending on frequency *f* with wave mode *A*. The scattered waves are described by $u_{B}^{\mathrm{SC}}\left(f,\gamma \right)$ and are measured at certain sensing points with sensing angle γ along a sensing circle with radius $r_{\mathrm{sen}}$. In Equation (1), | · | defines the absolute value. The first kind of Hankel function of the first order $H_{1}^{\left(1\right)}\left(\xi_{B}\left(f\right)r_{\mathrm{sen}}\right)$ describes the propagation of the scattered waves from the origin, i.e., the point source at the damage center position, to the sensing point with mode *B* using the wavenumber $\xi_{B}\left(f\right)$. To capture only the propagating wave components generated by the complex wave damage interaction, the sensing circle needs to be outside the near field of the scattered wavefield. Typically, this near field ends already several plate thicknesses away from the damage [61].

Generally, the WDICs, i.e., the amplitude coefficients in Equation (1), also cover possible mode conversion phenomena. This means that an incident wave with mode *A* is scattered to a different wave mode *B*, e.g., an incident wave with fundamental symmetric $S_{0}$ mode is converted to a scattered wave with fundamental asymmetric wave mode $A_{0}$ or vice versa. However, this study focuses on the wave damage interaction for the direct scattering from an incident $A_{0}$ to a scattered $A_{0}$ wave mode, i.e., $A=B=A_{0}$. This can be explained by two reasons. First, the experimental measurements of guided waves with the fundamental asymmetric $A_{0}$ wave mode using a single SLDV gives better results due to its reasonable out-of-plane wave motion. Second, its wavelength is smaller in comparison to the symmetric $S_{0}$ mode, and this increases the sensitivity to small damages. Hence, for simplicity, from now on, the amplitude coefficients $C_{A_{0}A_{0}}$ are abbreviated as *C*. The phase information of the generally complex-valued WDICs, i.e., the phase coefficients, could be additionally integrated in the proposed methodology for damage identification. However, the phase coefficients and their integration are not presented herein for the conciseness of the discussion.

For guided wave applications, the effect of possible damage is typically evaluated by analyzing the scattered wavefield, which is isolated by the baseline subtraction method [18]. Therefore, the scattered wave displacement $u^{\mathrm{SC}}$ is calculated by

(2) $$\begin{array}{c} u^{\mathrm{SC}}=u^{\mathrm{TOT}}-u^{\mathrm{IN}} \end{array}$$

where $u^{\mathrm{IN}}$ is the incident wave displacement in the pristine scenario and $u^{\mathrm{TOT}}$ is the total wave displacement in the damaged scenario. Furthermore, these wavefields can be decomposed by

(3) $$\begin{array}{c} \begin{array}{cc} u_{S_{0}} & =\frac{u_{\mathrm{Top}}-u_{\mathrm{Bot}}}{2}\\ u_{A_{0}} & =\frac{u_{\mathrm{Top}}+u_{\mathrm{Bot}}}{2} \end{array} \end{array}$$

in the fundamental symmetric $S_{0}$ and asymmetric $A_{0}$ wave modes. In Equation (3), $u_{\mathrm{Top}}$ and $u_{\mathrm{Bot}}$ are the out-of-plane displacements at the top and bottom sides of the structure, respectively. It is to say that Equations (1)–(3) can also be used for given out-of-plane velocities *v*, e.g., experimental SLDV measurements, by substituting the out-of-plane displacements *u*.

In general, the scattering of the incident waves by the damage might also generate higher guided wave modes. The WDICs can describe such higher-mode conversion phenomena. This requires a more sophisticated decomposition of the wavefields based on the varying mode shapes in the thickness direction [62]. However, in the present study, only the fundamental guided wave modes exist in the selected frequency range for the investigated structure. Hence, the elementary wave decomposition in Equation (3) is sufficient.

### 2.2. Deep Neural Networks

**Figure 2:**
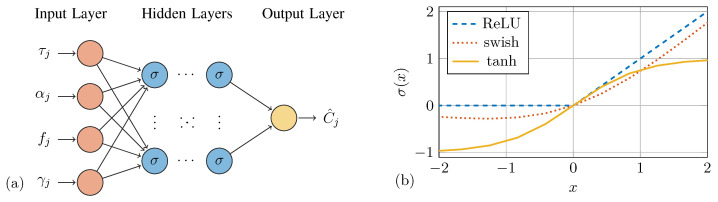
Deep neural network modeling: (**a**) architecture; (**b**) selected non-linear activation functions [50].

Complex relationships between variables can be learned from data using DNNs. They are non-linear models inspired by biological neural systems. The present study considers only deep feed-forward neural networks with an input layer of four neurons. This allows for passing an input vector $z_{0,j}={\left[f_{j},\gamma_{j},\tau_{j},\alpha_{j}\right]}^{T}$ containing a given frequency $f_{j}$, sensing angle $\gamma_{j}$, thickness ratio $\tau_{j}$ and orientation $\alpha_{j}$ to the DNN for predicting a scalar WDIC amplitude $z_{l,j}={\hat{C}}_{j}$. The ^ denotes DNN prediction. The four input features were selected based on their direct physical relevance to the problem, which ensures an informative feature space. The output layer thus contains only a single neuron for solving the corresponding regression problem. Figure 2a shows this architecture as a graph of interconnected neurons.

The model can be mathematically stated as

(4) $$z_{l,j}=M_{l}\left(M_{l-1}\left(\ldots M_{1}\left(z_{0,j}\right)\ldots \right)\right)$$

where $M_{i}$ is the affine mapping for the layers i=1…l. The mapping $M_{i}$ from the (i−1)-th layer with vector $z_{i-1,j}$ to the *i*-th layer is given as

(5) $$M_{i}\left(z_{i-1,j}\right)=z_{i,j}=\sigma_{i}\left(W_{i}z_{i-1,j}+b_{i}\right)$$

where $W_{i}$ is the $\left(m_{i}\times m_{i-1}\right)$-dimensional weight matrix and $b_{i}$ is the $\left(m_{i}\times 1\right)$-dimensional bias vector. The activation function of layer *i* is denoted by $\sigma_{i}\left(\cdot \right)$, which is applied element-wise. By using non-linear functions, a DNN becomes a non-linear model. The output $z_{i,j}$ of the *i*-th layer is, therefore, a non-linearly transformed weighted sum of its input. The selection of activation functions was motivated by their respective advantages: ReLU mitigates vanishing gradient issues, swish offers smooth non-linearity, and the hyperbolic tangent function provides symmetric output ranges; see Figure 2b. It should be noted that the output layer uses the identity function as an activation function to reproduce unrestricted sets as required for regression tasks.

The actual values of the model parameters, i.e., weights and biases, are obtained by so-called training. This is an optimization procedure that minimizes a loss function. The mean squared error (MSE) is commonly utilized for regression problems. The MSE can be calculated for $n_{\mathrm{samp}}$ training samples by

(6) $$\mathrm{MSE}=\frac{1}{n_{\mathrm{samp}}}\sum_{j=1}^{n_{\mathrm{samp}}}{\left({\hat{z}}_{l,j}-z_{l,j}\right)}^{2}$$

where ${\hat{z}}_{l,j}$ and $z_{l,j}$ are the *j*-th samples of the estimated and true model outputs, respectively. Common optimization algorithms are based on gradient descent minimization and mini-batch training. In the present study, stochastic gradient descent (SGD), RMSprop, AdaGrad and Adam are considered. The root mean square error (RMSE), defined as

(7) $$\mathrm{RMSE}=\sqrt{\mathrm{MSE}}$$

is a common metric for evaluating the final performance of regression models. Lower values indicate smaller differences between the predicted and true model outputs.

Furthermore, the mean absolute error (MAE) is utilized to evaluate the performances of selected models. The MAE is defined by

(8) $$\mathrm{MAE}=\frac{1}{n_{\mathrm{samp}}}\sum_{j=1}^{n_{\mathrm{samp}}}\left|{\hat{z}}_{l,j}-z_{l,j}\right|$$

and calculates the sum of absolute errors, i.e., the L1-norm, normalized by the sample size $n_{\mathrm{samp}}$. Analogous to RMSE, lower values signify a closer match between the predicted and true outputs of the model. Additionally, the coefficient of determination $R^{2}$ serves as a statistical measure of model performance. It quantifies the variance of the model prediction errors in comparison to the total variance of the true outputs and can be calculated by

(9) $$R^{2}=1-\frac{\sum_{j=1}^{n_{\mathrm{samp}}}{\left(z_{l,j}-{\hat{z}}_{l,j}\right)}^{2}}{\sum_{j=1}^{n_{\mathrm{samp}}}{\left(z_{l,j}-{\bar{z}}_{l}\right)}^{2}}\;$$

where ${\bar{z}}_{l}$ is the mean of the true model outputs. An $R^{2}$ value of 0.0 implies that the model performs no better than simply predicting the mean of the outputs, while $R^{2}<0.0$ indicates that the model underperforms compared to simply using the mean value as a predictor. The maximum of $R^{2}=1.0$ describes an ideal model that perfectly predicts the true outputs without error.

**Figure 3:**
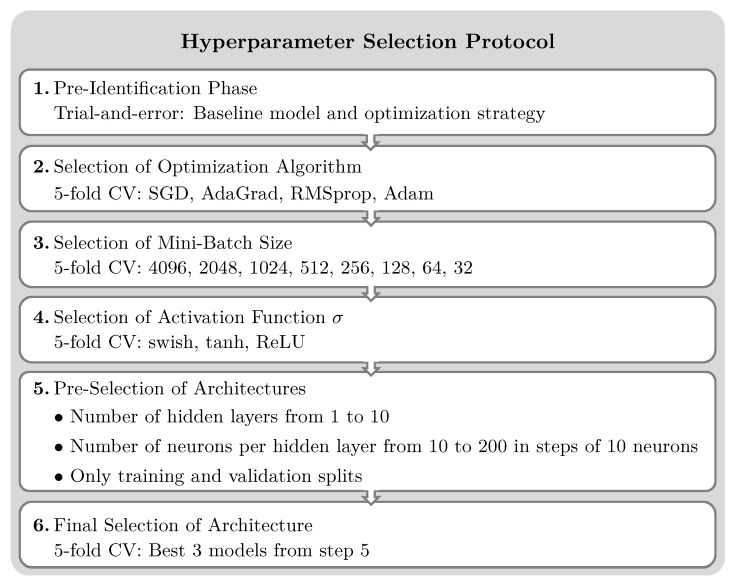
Cross-validation-based protocol for selection of DNN hyperparameters (based on [50]).

However, for obtaining an optimal performance in the present application, several hyperparameters need to be identified. The model architecture entails the selection of the number of layers and neurons per layer as well as the type of activation functions. Additionally, hyperparameters of the optimization procedure, i.e., the algorithm and the mini-batch size, are required. A hyperparameter selection protocol introduced in [50] is employed for this task; see Figure 3.

It is a sequential procedure starting from an initial trial-and-error phase for obtaining a baseline model and optimization strategy. Next, the optimization algorithm, the mini-batch size and the activation function are varied one after another. For a reliable selection, *k*-fold cross-validation is employed. For this task, a statistically motivated model selection criterion, ${\hat{\eta }}_{k-1,1-\beta }$, is defined:

(10) $${\hat{\eta }}_{k-1,1-\beta }={\bar{\mathrm{MSE}}}_{\mathrm{val}}+s_{{\mathrm{MSE}}_{\mathrm{val}}}t_{k-1,1-\beta }\;$$

where ${\bar{\mathrm{MSE}}}_{\mathrm{val}}$ and $s_{{\mathrm{MSE}}_{\mathrm{val}}}$ are the mean and standard deviation of the MSE estimated from the unseen cross-validation estimates. The minimal value indicates the optimal parameter. Then, the model architecture, i.e., number of layers and neurons per layer, is pre-selected using only a single hold-out cross-validation with lower computation burdens due to the high number of model candidates. The final architecture is obtained by *k*-fold cross-validation of the best three models obtained in the previous step. This final model architecture is then trained with early stopping and the available data using the selected stochastic optimizer followed by optimization with the help of the limited-memory Broyden–Fletcher–Goldfarb–Shanno (L-BFGS) optimizer, as a quasi-Newton method, for reaching the optimum faster. It should be noted that the exclusive use of the latter optimizer did not allow for learning the WDIC patterns.

The results of the hyperparameter selection procedure with the final architecture are presented in the Deep Neural Network Modeling Section. The final DNN enables predicting a scalar WDIC amplitude $\hat{C}\left(f,\gamma ,\alpha ,\tau \right)$ for a given frequency *f*, sensing angle γ, thickness ratio τ and orientation α, where the range of input values should be within the training data for reliable results. However, the geometrical scalability of WDICs increases the applicability for a large number of geometrical similar structures [49]. Furthermore, it should be noted that the selected architecture enables interpolation not only between different damage scenarios but also for frequencies and angles. This means that WDICs can be predicted for any angle, and the frequency resolution can be changed according to the task without additional training. Finally, in a previous study [50], it was found that other machine learning algorithms, i.e., support vectors, k-nearest neighbors and random forests, do not enable an interpolation between the training data at all or result in lower performance. Thus, only the discussed DNN approach is considered herein.

### 2.3. Anglewise Principal Component Analysis

High feature dimensionality and uncertainties due to the limited availability of potentially noisy training data can adversely affect the performance of an SHM algorithm [29]. To reduce such effects, PCA is a common technique. For the present application, an anglewise PCA of WDICs of a large number of predicted damage scenarios is proposed, i.e., selected combinations of thickness ratios τ and damage orientations α.

The WDICs are inherently represented in polar coordinates to describe the angular dependencies of the wave damage interaction patterns. To preserve these angular dependencies, the PCA is calculated individually for each requested sensing angle γ. This anglewise approach ensures optimal feature compression by tailoring the dimensionality reduction to the specific characteristics of the WDICs at each sensing angle. Consequently, the number of principal components (PCs) required for each sensing direction can vary and is not known beforehand. This variability allows the proposed anglewise PCA to adapt to the unique complexity of the WDICs for each angle, ensuring both computational efficiency and accuracy. An individual PCA can be performed for each requested sensing angle γ as follows.

First, WDICs are predicted by the DNN for a selected sensing angle. The vector of $n_{f}$ frequencies $f=\left[f_{1},f_{2},\ldots ,f_{n_{f}}\right]$ is kept fixed for each damage scenario *d* to obtain a single frequency line, i.e., the amplitude coefficient vector $c_{d}$, given as

(11) $${\hat{c}}_{d,\gamma }={\left[\begin{array}{cccc} \hat{C}\left(f_{1},\gamma ,\alpha_{d},\tau_{d}\right) & \hat{C}\left(f_{2},\gamma ,\alpha_{d},\tau_{d}\right) & \cdots & \hat{C}\left(f_{n_{f}},\gamma ,\alpha_{d},\tau_{d}\right) \end{array}\right]}^{T}$$

where $\hat{C}\left(f,\gamma ,\alpha_{d},\tau_{d}\right)$ is a scalar WDIC prediction. In the following, the sensing angle γ and the denomination of DNN predictions by ^ are omitted for the sake of simplicity. The frequencies are defined by the damage identification application, where the resolution can be different from the training data set. Due to the interpolation capabilities and computational efficiency of the proposed DNNs, such amplitude coefficient vectors $c_{d}$ can be calculated ad hoc for a large number of damage scenarios to construct a WDIC matrix C of dimension $\left(n_{f}\times n_{d}\right)$ as

(12) $$C=\left[\begin{array}{cccc} c_{1} & c_{2} & \ldots & c_{n_{d}} \end{array}\right].$$

Second, a centered WDIC matrix $C^{*}$ can be obtained by removing the estimated mean $\tilde{c}$ of the frequency lines from each column as

(13) $$C^{*}=\left[\begin{array}{cccc} c_{1}-\tilde{c} & c_{2}-\tilde{c} & \ldots & c_{n_{d}}-\tilde{c} \end{array}\right]$$

where

(14) $$\tilde{c}=\frac{1}{n_{d}}\sum_{d=1}^{n_{d}}c_{d}.$$

The variance–covariance matrix $\Sigma_{C^{*}}$ is estimated from the centered WDIC matrix $C^{*}$ by

(15) $$\Sigma_{C^{*}}=\frac{1}{n_{d}-1}C^{*}C^{*T}.$$

and can be expressed as an eigenvalue decomposition

(16) $$\Sigma_{C^{*}}=T_{\mathrm{PC}}\Lambda T_{\mathrm{PC}}^{T}$$

where $T_{\mathrm{PC}}$ is the PCA transformation matrix and the diagonal matrix Λ contains the eigenvalues of $\Sigma_{C^{*}}$ arranged in descending order by the variance of the corresponding PCA scores. The PCs are the normalized eigenvectors stored as column vectors in $T_{\mathrm{PC}}$. Fourth, the obtained PCs allow for calculating the PCA scores $C_{\mathrm{PC}}$ of the initial features by a linear transformation as

(17) $$C_{\mathrm{PC}}=T_{\mathrm{PC}}^{T}C$$

where the resulting features/scores $C_{\mathrm{PC}}$ are linearly independent and their variances are maximized in the first few dimensions. The score matrix $C_{\mathrm{PC}}$ contains the PCA scores as its columns.

Finally, by selecting PCs, the performance of an SHM algorithm can be improved and, at the same time, the feature dimensionality can be reduced. A common quantity for choosing PCs is the explained variance or other related variables, such as the eigenvalues, because this allows for maximizing the variability of a data set in a few dimensions. However, this is not necessarily optimal for damage identification because variations not related to damage, such as noise, may reduce the feature sensitivity [63]. Therefore, the Pearson correlation coefficient between scalar scores and values of a certain damage characteristic is employed as a selection criterion, where a higher absolute value of the correlation coefficient indicates a more sensitive feature. For example, the Pearson correlation coefficient $r_{{\mathrm{PC}}_{j},\tau }$ between the scores of the *j*-th PC $C_{{\mathrm{PC}}_{j}}$ and the thickness ratio τ can be calculated over $n_{d}$ damage scenarios as

(18) $$r_{{\mathrm{PC}}_{j},\tau }=\frac{\sum_{d=1}^{n_{d}}\left(C_{{\mathrm{PC}}_{j},d}-{\tilde{C}}_{{\mathrm{PC}}_{j}}\right)\left(\tau_{d}-\tilde{\tau }\right)}{\sqrt{\sum_{d=1}^{n_{d}}{\left(C_{{\mathrm{PC}}_{j},d}-{\tilde{C}}_{{\mathrm{PC}}_{j}}\right)}^{2}}\sqrt{\sum_{d=1}^{n_{d}}{\left(\tau_{d}-\tilde{\tau }\right)}^{2}}}$$

where the ˜ indicates the estimated mean. The Pearson correlation coefficient is bounded between ±1, where higher absolute values indicate higher linear correlation. The PCs with $|r_{{\mathrm{PC}}_{j},\tau }|\geq r_{\mathrm{req}}$ are selected for calculating scores with improved sensitivity. This can be similarly performed for the damage orientation α or other damage characteristics. For damage identification, the union of all selected PC subsets can be used to obtain scores of the DNN-predicted WDICs as well as the measured WDICs. This allows for accounting for the rotational WDIC characteristics and sensitivities concerning damage.

**Figure 4:**
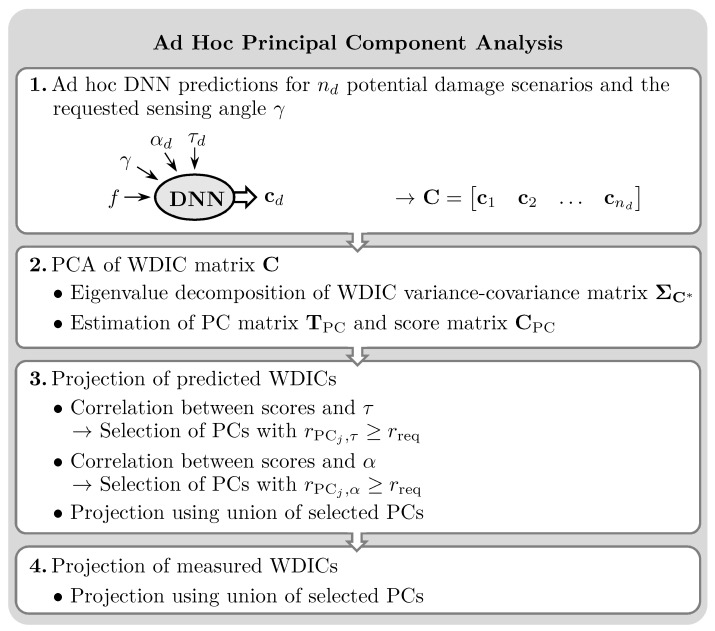
Flowchart for ad hoc PCA for feature compression (sensing angle and denomination for DNN prediction by ^ are omitted).

The flowchart in Figure 4 summarizes the calculation steps for the ad hoc anglewise PCA for feature compression. These compressed feature vectors can be used analogously in the proposed damage identification method. Therefore, the anglewise PCA can be inserted as an additional step in the online identification phase of the presented methodology; see Figure 1. The length of the compressed feature vector results from the number of selected PCs, which depend on the sensing angle. Therefore, the PCA is calculated from the ad hoc WDIC predictions for exactly the requested sensing angles, which are previously unknown in the offline training phase. However, the WDIC predictions are required anyway by the proposed damage identification technique. Thus, the additional computation time for performing PCA is negligible.

### 2.4. Damage Identification by Ad Hoc Predictions

Generally, WDICs are highly sensitive damage features and depend on the characteristics of the damage, e.g., size, orientation and type. This means that any changes in these damage characteristics cause variations in the WDIC scattering pattern in angular direction as well as over frequency, which stimulates the idea of a WDIC-based damage identification approach [50]. In practical applications, the damage is detected and localized prior to the identification procedure. Anomaly detection [19] or the baseline subtraction method [18] can be used to indicate the existence of damage. The latter reveals the scattered waves caused by the damage. Further analysis, e.g., by time-of-flight [54] or time-reversal approaches [52], enables us to estimate the damage position from the wave scattering. These localization procedures require at least three different actuator–sensor pairs for a unique result. The positions of the actuator and the sensors related to the estimated damage position determine the sensing angles $\gamma_{1},\gamma_{2}$ and $\gamma_{3}$, which are used for damage identification. For each sensing angle $\gamma_{i}$, the amplitude coefficient vector $c_{\gamma_{i}}$ of the unknown damage is calculated for the frequency vector $f=\left[f_{1},f_{2},\ldots ,f_{n_{f}}\right]$ from the measured scattered wave signals using Equation (1). They are then combined into a single feature vector $c^{\mathrm{st}}$ for the unknown damage with dimensions $\left(3n_{f}\times 1\right)$ as

(19) $$\begin{array}{c} c^{\mathrm{st}}={\left[\begin{array}{ccc} c_{\gamma_{1}}^{T} & c_{\gamma_{2}}^{T} & c_{\gamma_{3}}^{T} \end{array}\right]}^{T}. \end{array}$$

The proposed damage identification method compares this feature vector to DNN predictions for the requested sensing angles, which can be analyzed ad hoc, i.e., during an inspection within a short time, directly on site. Therefore, the previously trained DNN predicts the amplitude coefficient vectors ${\hat{c}}_{d,\gamma_{i}}$ for every sensing angle $\gamma_{i}$, which are combined (analogously to $c^{\mathrm{st}}$ in Equation (19)) into a single feature vector ${\hat{c}}_{d}^{\mathrm{st}}={\left[\begin{array}{ccc} {\hat{c}}_{d,\gamma_{1}}^{T} & {\hat{c}}_{d,\gamma_{2}}^{T} & {\hat{c}}_{d,\gamma_{3}}^{T} \end{array}\right]}^{T}$ for each damage scenario *d*. According to Humer et al. [50], the damage metric $M_{d}$ is selected for damage identification as

(20) $$\begin{array}{c} M_{d}={∥{\hat{c}}_{d}^{\mathrm{st}}-c^{\mathrm{st}}∥}_{1} \end{array}$$

and calculates the L1-norm (Manhattan distance) between the feature vectors of the DNN predictions ${\hat{c}}_{d}^{\mathrm{st}}$ for each damage scenario *d* and the feature vector $c^{\mathrm{st}}$ measured from the unknown damage. Small values of the damage metric $M_{d}$ represent small differences between the feature vectors. Hence, the minimal value of the damage metric indicates the identified damage scenario $d^{*}$ and its properties, i.e., thickness ratio $\tau_{d^{*}}$ and orientation $\alpha_{d^{*}}$.

In the present study, the performance of the proposed damage identification method is tested additionally using an anglewise PCA for feature compression. Therefore, the amplitude coefficient vectors in the feature vector are replaced by their scores calculated by PCA for each requested sensing angle used for identification. This feature compression is applied to the DNN’s predicted feature vector ${\hat{c}}_{d}^{\mathrm{st}}$ for all damage scenarios *d* as well as to the measured feature vector of the unknown damage $c^{\mathrm{st}}$. Thus, the dimensions of the resulting feature vectors are reduced significantly. Due to the dependency of PCA on the sensing angle, the calculations are performed ad hoc for the requested sensing angles.

## 3. Application

The performance of the proposed damage identification method is investigated for a simulation-based and an experimental data-driven configuration. In this section, the underlying physical experiments and numerical simulations are explained in detail. Furthermore, the subsequent implementation of the DNN modeling and anglewise PCA are described.

### 3.1. Physical Experiments

**Figure 5:**
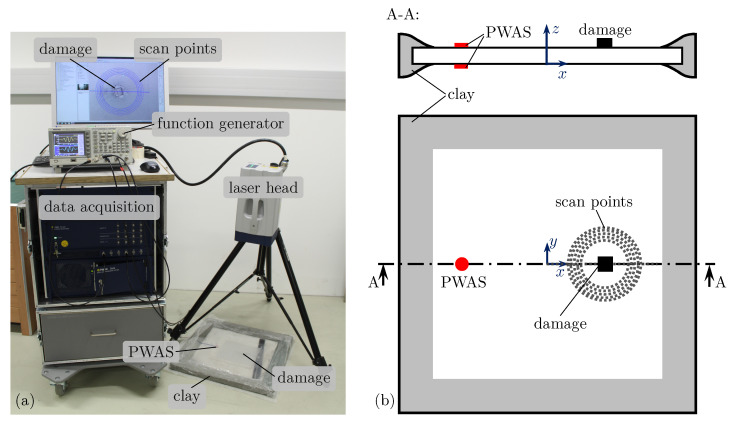
Physical experiments: (**a**) SLDV measurement setup; (**b**) sketch [49].

A detailed description of the physical experiments can be found in [49]. The corresponding data are reused in this study. Hence, only the main facts of the experimental setup are summarized. However, the investigated specimen is a 2mm thin aluminum alloy (EN AW-5754 [64]) plate with a size of $500\times 500\;{\mathrm{mm}}^{2}$. In this plate, only the fundamental symmetric $S_{0}$ and asymmetric $A_{0}$ guided wave modes exist for the examined frequencies below 300kHz. Generally, the out-of-plane motion of the fundamental asymmetric $A_{0}$ wave mode is more dominant compared to the fundamental symmetric $S_{0}$ mode. The out-of-plane velocities of the guided waves are measured without contact by a single SLDV (Polytech PSV-500-HV [65]). Thus, the focus of this study is on the fundamental asymmetric $A_{0}$ wave mode. However, the investigated plate is equipped with two circular PWASs (PIC 255 [66]) with a diameter of ⌀10mm and a thickness of 0.2mm to excite guided waves in the structure. They are attached at the position x=−100mm on the top and bottom surfaces of the plate by structural epoxy as depicted in Figure 5b. The SLDV is used to assess many different sensor locations (sensing angles $\gamma_{i}$) with reasonable experimental demands instead of moving the damage. Nevertheless, in a real situation, fixed PWAS sensors can be used for identifying damages in different locations.

An arbitrary function generator supplies them directly with a five-period von Hann windowed sine function with an amplitude of 9V. Due to the selected arrangement of the PWASs opposite to each other and asymmetrical electrical excitation, i.e., the bottom PWAS is excited with the inverted signal of the top one, the asymmetric $A_{0}$ mode is excited predominantly. To increase the usable frequency range for WDIC evaluation, two burst signals with overlapping frequency spectra are used in successive experiments. Hence, the experimental results from excitation signals with central frequencies of $f_{c}=130\;\mathrm{kHz}$ and $f_{c}=200\;\mathrm{kHz}$ are combined.

Further, miniature steel sheets with quadratic shape and an edge length of 12.5mm are bonded to the top surface of the plate as reversible structural changes, i.e., artificial damages, using regular superglue. Such artificial damages are simple to remove or reapply, e.g., at different positions or with different orientations α, which is common practice in experimental laboratory measurements [51,52]. However, in this study, the damage position $x_{D}=50\;\mathrm{mm}$ is fixed and the influence of a varying damage thickness ratio τ and orientation α on the WDIC pattern is investigated. Therefore, measurement points every $2.5^{\circ }$ along a sensing circle (centered at the damage position) with radius $r_{\mathrm{Sen}}=30\;\mathrm{mm}$ are measured by an SLDV; see Figure 5a. In addition to the wave damage interaction, the guided waves are reflected at the boundaries of the plate. The edges of the plate are covered by specifically molded clay to suppress disturbances by these reflections.

In general, damage scatters the incident-guided waves in different wave modes with varying intensities [62]. To account for such mode conversion phenomena, the incident and scattered waves are decomposed in their parts, i.e., the symmetric $S_{0}$ and asymmetric $A_{0}$ modes. Therefore, the SLDV measures the guided wavefields from the bottom and the top sides of the plate in subsequent experiments. Using Equation (3) enables us to adjust the guided wave modes. This requires a realignment of the SLDV in relation to both sides of the plate, which might cause a systematic positioning error. Thus, Humer et al. [49] proposed an improved baseline subtraction to reduce the effect of such positioning errors on the measurement signals. However, after further data processing steps, e.g., bandpass filtering, time history tapering, etc., the WDIC amplitudes can be calculated using Equation (1). These steps are not presented in detail here to not distract the reader and can be found in [49].

**Figure 6:**
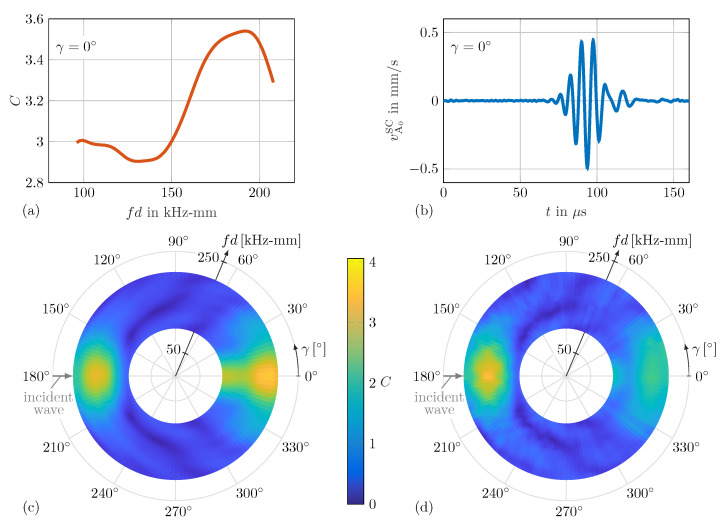
Numerical and experimental results ($\alpha =0^{\circ }$ and τ=1): (**a**) numerical simulation results; (**b**) typical SLDV measurement signal; (**c**) numerical WDIC pattern; (**d**) experimental WDIC pattern.

A typical SLDV measurement signal for the scattered asymmetric wave velocity $v_{A_{0}}^{\mathrm{SC}}$ is depicted in Figure 6b. The resulting WDIC pattern for the scan along the sensing circle can be seen in Figure 6d. This color-coded representation for the amplitude coefficients *C* allows us to visualize their dependencies on varying damage characteristics for both the sensing angle γ and the characteristic product of frequency *f* and half plate thickness *d* at once.

### 3.2. Numerical Simulations

The CAFA combines analytical considerations and numerical simulation results to a semi-analytical method, which can describe the scattering of guided waves by arbitrarily shaped damages [48]. Therefore, a specifically designed FE model is used to extract WDICs directly in the frequency domain. The numerically simulated WDIC data presented herein are reused from previous studies [49,50]. However, the implementation of the FE model in the commercial simulation software ABAQUS 2018 [67] is described in the following.

The investigated structure is a 1.6mm thin aluminum plate. The artificial damages are miniature steel sheets bonded to the top surface of the plate with different orientations α and thickness ratios τ; see Table 1. They have a square shape with an edge length D=10mm. The material properties for the aluminum plate and the steel damages are summarized in Table 2.

**Figure 7:**
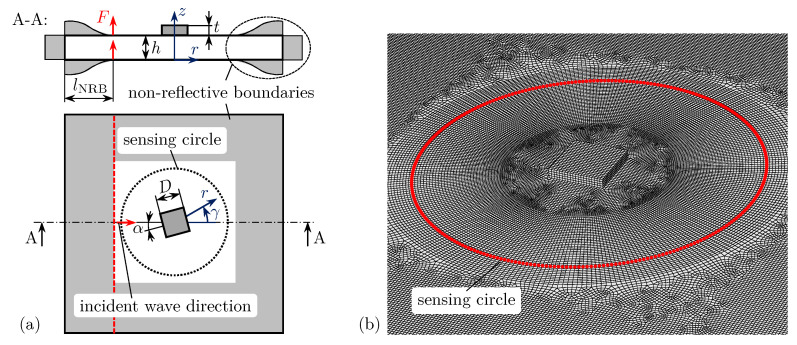
Local FE model: (**a**) sketch; (**b**) center region mesh with highlighted sensing nodes [49].

The bonding between the damage and the plate is assumed to be ideal. Therefore, the mesh of the damage and the plate matches in the contact region, where the nodes are connected using a tie connection. For simulating wave propagation phenomenons, the spatial discretization, i.e., the mesh size, is crucial [68,69]. Hence, the plate is discretized with a maximum in-plane mesh size of 0.5mm, while four elements are used along the plate thickness; see Figure 7b. The FE model uses non-reflective boundaries to dampen the unwanted reflections from the plate boundaries, i.e., an infinitely large plate is simulated [70]. This allows for using steady-state dynamics analysis with harmonic excitation to simulate the WDICs directly in the frequency domain. The non-reflective boundaries are applied over a length $l_{\mathrm{NRB}}=30\;\mathrm{mm}$ using a damping coefficient ζ=0.3 for the half von Hann window function following the recommendations in [70]; see Figure 7a. The resulting FE model with dimensions $150\times 150\times 1.6\;{\mathrm{mm}}^{3}$ contains approximately 810,000 elements. The selected linear solid element type C3D8 uses full integration. The numerical results are calculated in a frequency range between 80kHz and 320kHz in steps of 2kHz, which needs approximately 7h using a standard desktop computer.

In practical SHM applications, e.g., using a sparse PWAS array, the distance between possible damage and the actuator PWAS is usually relatively large in comparison to typical damage sizes. Hence, the curvature of the incident wave is neglected in this simulation model. Two straight-line loads on the top and bottom of the plate (on the left side of the damage) are asymmetrically excited to generate straight-crested guided waves with an asymmetric $A_{0}$ mode; see Figure 7a. Generally, a pair of simulations (one for the pristine and one for the damaged scenario) is required for the analysis of the scattered wavefield generated by the wave damage interaction. For both cases, the out-of-plane nodal displacements are extracted every $1.25^{\circ }$ along the sensing circles with radius $r_{\mathrm{sen}}=30\;\mathrm{mm}$ on the top and bottom of the plate; see Figure 7. From these results, the scattered wave displacements are isolated and decomposed by using Equations (2) and (3), resulting in $u_{A_{0}}^{\mathrm{SC}}$ and $u_{S_{0}}^{\mathrm{SC}}$, respectively. Analogously, the incident wave displacement $u_{A_{0}}^{\mathrm{IN}}$ is evaluated using the results of the pristine model for the top and bottom nodes at the equivalent damage center position. By inserting the resulting displacements $u_{A_{0}}^{\mathrm{SC}}$ and $u_{A_{0}}^{\mathrm{IN}}$ of the scattered and incident waves, respectively, in Equation (1), the WDICs can be calculated for twelve selected damages; see Table 1. For example, the numerical simulation results for damage No. 9 ($\alpha =0^{\circ }$ and τ=1) are shown in Figure 6a,c.

In general, the properties of the damage and the host structure influence the WDIC pattern. In this study, the latter does not change, and only the dependencies of the WDICs on the damage properties, i.e., the orientation α and dimensionless thickness ratio τ, are assessed. However, an extension of the numerical study would easily be possible.

### 3.3. WDIC Comparison

The selected dimensionless damage parameters, i.e., the damage-to-plate thickness ratio $\tau =\frac{t}{h}$, the damage size-to-thickness ratio $\delta =\frac{D}{t}$ and damage size-to-wavelength ratio $\Lambda =\frac{D}{\lambda_{A_{0}}}$, allow us to directly compare WDICs over the characteristic product of frequency *f* and half plate thickness *d* of differently sized but geometrically similar damages; see Table 3. For example, Figure 6c,d show the direct comparison of the numerical and experimental WDIC patterns for damage No. 9 (see Table 1). Generally, the scattering patterns show good agreement, and particularly the position and shape of the valleys, i.e., low values for the amplitude coefficients *C* (dark blue color), match closely. The values near the transmitted direction, i.e., sensing angle $\gamma =0^{\circ }$, show stronger deviations. This can be explained mainly by the neglected adhesive layer between the artificial damage and the host structure in the numerical model [49]. However, the influences of these differences on the damage identification performance using a simulation-based approach is discussed in Section 4.2 in detail.

### 3.4. Deep Neural Network Modeling

The purpose of using DNNs in the present research is the ability to interpolate complex WDIC patterns for different damage characteristics. This allows for making predictions for a large number of unseen damage scenarios as required for precise damage identification. For the experiment, the WDICs of the damage scenarios given in Table 1 are employed. The resulting training data set contains 196,620 samples of input vectors $z_{0,j}={\left[f_{j},\gamma_{j},\tau_{j},\alpha_{j}\right]}^{T}$. To improve the learning process, the inputs are normalized to the range of ±1 and the output samples are standardized before random shuffling. The pre-processing is performed with the Python package scikit-learn (version 0.20) [71]. The DNNs are created and optimized in Python using TensorFlow [30].

**Figure 8:**
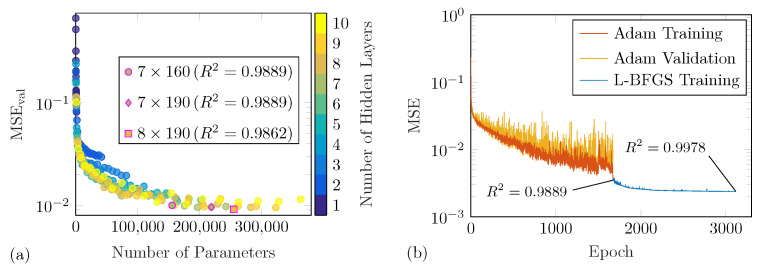
Mean squared errors: (**a**) different experimental DNN architectures for training up to 500 epochs (log scale); (**b**) final training of selected 7×160 experimental DNN using all data sets (log scale). The $R^{2}$ values are provided as additional information and are computed for all 12 training damage scenarios with models that share identical hyperparameters.

By adopting the hyperparameter selection protocol (see Section 2.2), the final hyperparameters were obtained from a baseline configuration of a 5×100 architecture with ReLU activation functions and the Adam optimizer with 128 samples. The mini-batch size was changed to 64, while the activation function and optimizer pre-selection were underpinned by the 5-fold cross-validation analysis steps. Next, a large number of candidate architectures was trained and evaluated by a simpler hold-out cross-validation. Figure 8a shows the obtained validation MSEs. It can be seen that with an increasing number of hidden layers and trainable model parameters the errors decrease. In addition to the model with minimal-validation MSE (8×190 with 255,171 parameters), models with 7×190 and 7×160 architectures and 218,881 as well as 155,521 parameters, respectively, were selected for a more reliable 5-fold cross-validation analysis. The 7×160 DNN was finally selected due to low mean values and the variability of the model selection criterion for the last 50 training epochs. Lastly, this model was trained using all experimental data and two-stage optimization; see Figure 8b.

**Figure 9:**
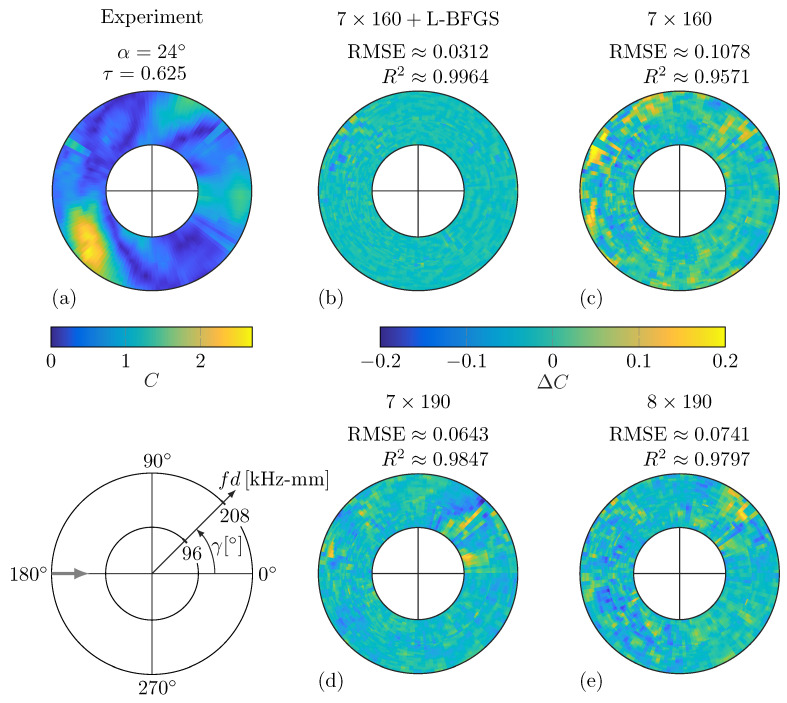
(**a**) Experimental measurement results (true values) for off-grid damage ($\alpha =24^{\circ }$ and τ=0.625, not contained in the training data sets) and WDIC differences ΔC between DNN predictions and true values for selected models: (**b**) 7×160 with L-BFGS optimization, (**c**) 7×160, (**d**) 7×190 and (**e**) 8×190 with corresponding RMSE and $R^{2}$ values from Table 4 (ΔC is limited between −0.2 and 0.2 for visualization).

In Table 4, the prediction accuracies of selected models are presented using the RMSE, MAE and $R^{2}$ metrics, as defined in Equations (7)–(9). It is important to note that these values are evaluated for the fully trained models, while Figure 8 illustrates data during the optimization process. These selected models share the same architecture and hyperparameters. However, the values in Table 4 demonstrate that the prediction accuracies are generally high for the individual damage scenarios as well as when calculated for all training data sets. The 7×160 model performs comparably well relative to the 7×190 and 8×190 models, despite having less parameters. Notably, the final L-BFGS optimization of the 7×160 model significantly improves the prediction accuracy. The off-grid damage scenario ($\alpha =24^{\circ }$ and τ=0.625) was not included in the training data sets, i.e., it was not available for model optimization or selection. Nonetheless, the DNN predictions for this damage case are remarkably accurate. For this off-grid damage scenario, the WDIC differences ΔC between the DNN predictions of the selected models and the true values are depicted in Figure 9 with corresponding RMSE and $R^{2}$ values from Table 4. In Figure 9b, the substantial improvement in the final L-BFGS training of the two-stage optimization can be seen.

The numerical DNN is reused from [50], where WDICs from twelve numerically simulated damage scenarios were utilized for DNN modeling. The hyperparameters were identified according to the same selection protocol employed for the experimental DNN. Just the set of potential architectures in Step 5 was different. Here, models with one to ten hidden layers of 10 to 100 neurons each and a few selected bottleneck architectures were studied. The latter did not provide any benefits compared to conventional models. Therefore, the final numerical DNN has a conventional architecture with 7×80 neurons using the swish activation function. The two-step training was performed using the Adam optimizer with 256 samples per mini-batch followed by the L-BFGS minimization. The final hyperparameters of both DNNs are presented in Table 5. The differences can be attributed to the characteristics of the training WDIC patterns, where the numerical WDICs are more smooth compared to the experimental ones. The latter appear scattered between frequency lines due to noise caused by the data acquisition. This explains the identification of a more complex experimental DNN with a discontinuous activation function, i.e., ReLU. It should be noted that the final RMSE of the experimental DNN with a value of 0.0488 is significantly higher than the 0.0055 achieved by the numerical DNN. Nevertheless, a visual inspection of predictions indicated a sufficient performance. Thus, it is employed for the following damage identification.

However, this study demonstrates that the proposed DNN modeling approach by Humer et al. [50] using a hyperparameter selection protocol is applicable to not only smooth WDIC patterns obtained by numerical simulations but also noise-affected experimental measurements, where WDICs can be similarly interpolated and predicted. Additionally, the geometrical scaling property of WDICs enables the reuse of trained DNNs for different but geometrical similar structures without additional effort; see Table 3. Furthermore, the selected DNN architecture accounts for potentially different frequency resolutions between the training and testing data.

### 3.5. Anglewise Principal Component Analysis

**Figure 10:**
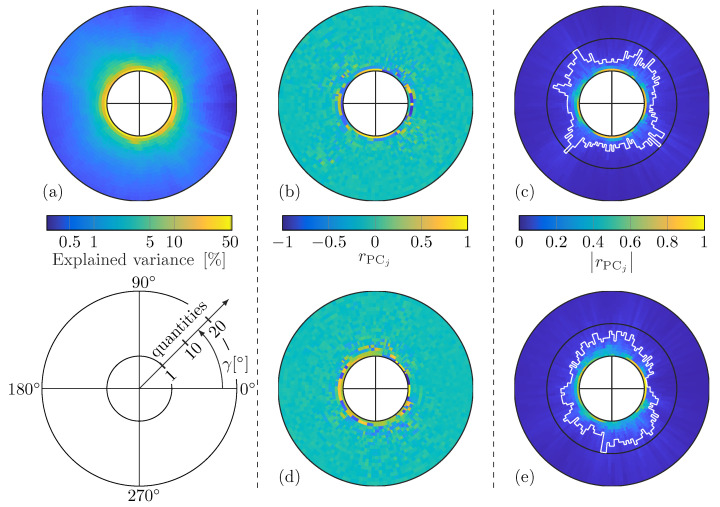
PCA results of experimental DNN predictions: (**a**) explained variance; (**b**) correlation coefficient $r_{{\mathrm{PC}}_{j},\alpha }$ for orientation α; (**c**) sorted absolute values of correlation coefficient $\left|r_{{\mathrm{PC}}_{j},\alpha }\right|$ for orientation α; (**d**) correlation coefficient $r_{{\mathrm{PC}}_{j},\tau }$ for thickness ratio τ; (**e**) sorted absolute values of correlation coefficient $\left|r_{{\mathrm{PC}}_{j},\tau }\right|$ for orientation τ.

PCA is a common technique to reduce feature dimensionality and can be applied as an optional step in the proposed damage identification procedure; see Figure 4. Generally, WDIC patterns are highly direction-dependent and, thus, change with the sensing angle γ. Therefore, the PCA is calculated anglewise, i.e., individually for a specific sensing angle. The sensing angles used for identification are determined by the damage localization results in the online identification phase; see Figure 1. Hence, the PCA is evaluated from the ad hoc WDIC predictions by a DNN for exactly these sensing angles, which are unknown in the offline training phase. However, the results of an anglewise PCA of the experimental DNN predictions for a fixed frequency vector $f={\left[96,97,\ldots ,208\right]}^{T}\;\mathrm{kHz}$ of dimension (113×1) and 9191 damage cases, i.e., all combinations of 101 damage thickness ratios $\tau \in \left\{0.5,0.505,\ldots ,1\right\}$ and 91 damage orientations $\alpha \in \left\{0^{\circ },0.5^{\circ },\ldots ,45^{\circ }\right\}$, are illustrated in Figure 10 for sensing angles in steps of $2.5^{\circ }$.

Generally, a strong directional dependency can be noticed, which underpins the necessity of the proposed anglewise ad hoc analysis for damage identification. However, the proportion of explained variance is depicted in Figure 10a and is a statistical measure to represent the amount of variation in a data set that can be assigned to each PC (eigenvector). It can be seen that its values already decrease rapidly in the depicted first 20 out of 113 total PCs (in the radial direction) for all sensing angles (in the angular direction). This indicates the potential for a tremendous feature reduction using only a small number of PCs while retaining most of the damage information from the data set.

However, in this study, the Pearson correlation coefficient is utilized as a criterion for selecting the PCs for calculating scores with improved sensitivity. Hence, the coefficients $r_{{\mathrm{PC}}_{j},\tau }$ and $r_{{\mathrm{PC}}_{j},\alpha }$ are calculated according to Equation (18) for the damage thickness ratio τ and the damage orientation α for the *j*-th PC, respectively. Their results are shown in Figure 10b,d for the first 20 PCs. To select the PCs for calculating scores with improved sensitivities, the absolute values of the correlation coefficients are sorted in descending order and compared to a required minimal value $r_{\mathrm{req}}$ separately, i.e., only PCs with $|r_{{\mathrm{PC}}_{j},\tau }|\;\geq \;r_{\mathrm{req}}$ and $|r_{{\mathrm{PC}}_{j},\alpha }|\;\geq \;r_{\mathrm{req}}$ are selected; see Figure 10c,e. The white line shows the number of PCs selected for each sensing angle γ for a required correlation coefficient $r_{\mathrm{req}}=0.1$. It can be seen that depending on the sensing angle, 1 to 11 PCs are selected out of the total 113 (black circles indicate 10). The union of the selected PC subsets relating to the damage thickness ratio and the damage orientation of a certain sensing angle are used to calculate the scores of the DNN predictions as well as the measured WDICs of the unknown damage in this direction. The dimension of the scores resulting from the anglewise PCA of the experiment-based DNN predictions is reduced to a length between a minimum of 3 and a maximum of 13 for a required correlation coefficient $r_{\mathrm{req}}=0.1$; see Table 6. Generally, the results of the anglewise PCA of the simulation-based DNN predictions show similar behavior. The number of scores selected for a required correlation coefficient $r_{\mathrm{req}}=0.1$ is slightly higher, i.e., a minimum of 5 and a maximum of 15 scores; see Table 6.

However, for the proposed damage identification method, the scores are obtained exactly for the estimated sensing angles. The resulting scores are combined to single feature vectors, i.e., ${\hat{c}}_{d}^{\mathrm{st}}$ for the predicted damage scenario *d* and $c^{\mathrm{st}}$ for the measurement of the unknown damage (cf. Equation (19)), with significantly reduced dimensions. For a required correlation coefficient $r_{\mathrm{req}}=0.1$, the compressed feature vectors have a length between 11 and 37 scores with an average of 22 entries for the experiment-based results and between 15 and 45 scores with an average of 29 entries for the simulation-based results (cf. Table 6). In comparison, the full feature vector has a constant length of 339 (containing three amplitude coefficient vectors of length 113) independent from the sensing angle vector. However, the compressed feature vectors can be inserted in the damage metric in Equation (20) to identify the damage properties of the unknown damage. It should be noted that the dimensions of the scores for the different sensing angles are generally not the same. However, the transformations are identical for each prediction and measurement, which allows for comparing the resulting stacked feature vectors.

## 4. Damage Identification

In this section, the performance of the proposed damage identification method is tested and discussed. Therefore, the WDIC patterns are predicted by two different DNNs trained either by numerical simulation or by experimental measurement data. Due to the generalization capabilities of these DNNs, the predictions of the WDICs can be interpolated in between the training data ranges. This means that the amplitude coefficients can be predicted at any arbitrary point in the design space, which is defined by the damage properties thickness ratio and orientation as well as the frequency range and the sensing angles. For the following discussion, both DNNs predict in total 9191 different damage cases, i.e., 101 thickness ratios $\tau \in \left\{0.5,0.505,\ldots ,1\right\}$ and 91 damage orientations $\alpha \in \left\{0^{\circ },0.5^{\circ },\ldots ,45^{\circ }\right\}$, in a frequency range $f\in \left\{96,97,\ldots ,208\right\}\;\mathrm{kHz}$. These predictions are compared to the measured WDICs of an unknown damage by the damage metric $M_{d}$ in Equation (20) for a given sensing angle vector γ. The present study employs 10,000 unique triplets of sensing angles using a $2.5^{\circ }$ grid for assessing the performance for a wide range of potential sensor and damage locations. By means of these variations for the damage properties and the sensing angles, a statistical analysis of the damage identification results is presented for different test cases. Moreover, the damage identification results using the feature compression based on PCA are analyzed.

### 4.1. Experiment-Based Approach

In this test case, the experimental measurements of twelve different damage scenarios (see Table 1) are utilized as training data for the experimental DNN (see Section 3.4). This experiment-based DNN predicts the feature vectors ${\hat{c}}_{d}^{\mathrm{st}}$ for $n_{d}=9191$ different damage cases. All these predictions in the data set S are compared to the feature vector $c^{\mathrm{st}}$ of an unknown damage by the damage metric $M_{d}$ for the given sensing angles. The smallest damage metric indicates the identified damage and, with it, its damage properties for each of the tested 10,000 unique sensing angle combinations. However, the proposed damage identification method is first tested for the measurements of damage No. 6 (see Table 1), which is contained in the training data of the experiment-based DNN. For this example, the technique indicates in 8669 out of the 10,000 sensor combinations, i.e., approximately 86.7%, the exact damage properties τ=0.75 and $\alpha =15^{\circ }$ from the 9191 predicted damage scenarios, whereas 99.61% of classifications fall in a range of τ=0.75±0.005 and $\alpha =15^{\circ }\pm 0.5^{\circ }$. These excellent identification results might be expected for damage cases contained in the training data set. Nevertheless, it indicates accurate DNN predictions even when using potentially noisy experimental measurement data for training. Furthermore, this underpins the high sensitivity of the WDICs utilized as damage features. Additionally, damage identification is performed using compressed feature vectors based on the anglewise PCA. In this case, only 7037 angle combinations or 70.37% are perfectly identified, while 97.73% of classifications lay in the range of τ=0.75±0.005 and $\alpha =15^{\circ }\pm 0.5^{\circ }$. Similar results are obtained for the remaining training damage scenarios. This is underpinned by the statistical summary given in Table 7 with almost perfect mean estimates $\bar{\alpha }$ and $\bar{\tau }$ as well as very small standard deviations $s_{\alpha }$ and $s_{\tau }$.

**Figure 11:**
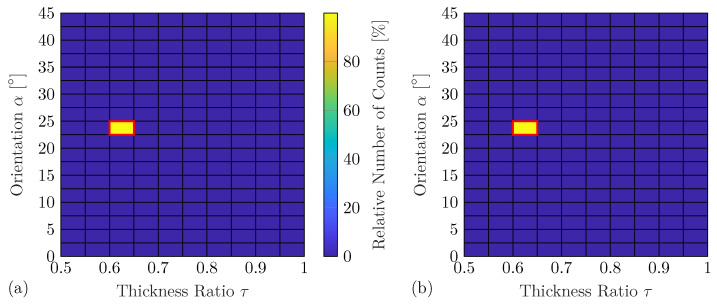
Damage identification results of unseen damage ($\alpha =24^{\circ }$τ=0.625; marked bin) based on experimental DNN predictions using (**a**) WDICs (full damage features); (**b**) scores of anglewise PCA (compressed damage features).

The next example represents a more realistic scenario in terms of a practical application of the damage identification method using damage with a thickness ratio τ=0.625 and orientation $\alpha =24^{\circ }$. This damage is not contained in the training data set of the DNN, and, thus, the interpolation accuracy of the WDIC predictions can be investigated. The technique indicates in 8582 out of the 10,000 sensor combinations, i.e., 85.82%, exactly the correct damage properties from the 9191 predicted damage scenarios. In the following, the intervals Δτ=0.05 and $\Delta \alpha =2.5^{\circ }$ are selected for visualization purposes, which results in 180 different damage scenarios. A previous study discusses the effect of different resolutions on the damage identification performance, where smaller intervals lead to decreasing correct classification rates [50]. However, Figure 11a shows that 99.93% of the samples fall in the bin of unseen damage. Comparably good results are obtained when using the compressed feature vectors based on anglewise PCA; see Figure 11b. The correct bin (τ=0.625±0.025 and $\alpha =22.5^{\circ }\pm 2.5^{\circ }$) contains 99.63% of the classifications. The statistical analysis summarized in Table 7 reveals that on average the correct damage properties are identified. For the reduced features, a significant increase in the standard deviations can be observed for both damage properties and over all damage scenarios in comparison to using the full feature vector. By using PCA, small differences between the predicted highly sensitive WDIC lines might become indistinguishable, which naturally increases the range of predicted properties.

However, the proposed damage identification method has an outstanding performance in a data-driven application when solely experimental measurement data are used. These results demonstrate the high accuracy of the smart interpolations and generalization capabilities of a DNN obtained by the hyperparameter selection protocol even for imperfect experimental training data.

### 4.2. Simulation-Based Approach

In this section, the proposed damage identification methodology is assessed in a simulation-based setting. Therefore, a DNN is created and trained solely by numerical simulation results of the twelve selected damage scenarios given in Table 1. Then, the experimental measurements according to these twelve training scenarios and additionally the unseen damage with a thickness ratio τ=0.625 and orientation $\alpha =24^{\circ }$ are used again for assessing the performance of the simulation-based DNN. Hence, this test case represents a possible application in a practical simulation-based SHM approach. However, the statistical analysis for this case using the full feature vector shows that the mean values are still within one standard deviation of the correct values for both damage properties α and τ; see Table 7. It is noticeable that the error of the mean values $\bar{\alpha }$ and $\bar{\tau }$ in relation to the correct values as well as the standard deviations $s_{\tau }$ and $s_{\alpha }$ are significantly increasing in comparison to the previous cases using the experiment-based DNN predictions. This might be explained by certain simplifications in the numerical FE model, e.g., the adhesive layer between the surface-bonded artificial damage and the host structure is neglected. Hence, the numerical and experimental WDIC patterns show differences; see Figure 6c,d. This enables us to investigate the influence of these common simplifications on the damage identification performance using the developed method. However, it is noticeable that for damages with orientation $\alpha =45^{\circ }$ the error for the mean estimates of the orientation $\bar{\alpha }$ are higher in comparison to the remaining orientations for all thickness ratios. This could be caused by a possibly too-coarse mesh near the corner regions of the damage in the FE model, which has a particularly high influence in this damage orientation.

For the simulation-based approach using the compressed feature vector, for the majority of the investigated damage cases, the mean values $\bar{\alpha }$ and $\bar{\tau }$ are marginally closer to the correct values for both damage properties α and τ. At the same time, the standard deviations $s_{\tau }$ and $s_{\alpha }$ tend to be slightly lower in comparison to the results using the full feature vector.

**Figure 12:**
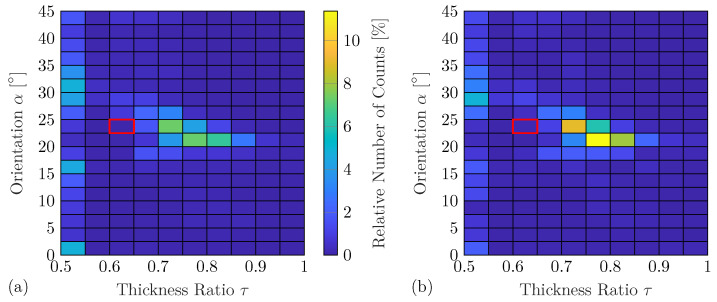
Damage identification results of unseen damage ($\alpha =24^{\circ }$τ=0.625; marked bin) based on simulation-based DNN predictions using (**a**) WDICs (full damage features); (**b**) scores of anglewise PCA (compressed damage features).

However, the following example again uses damage with a thickness ratio τ=0.625 and an orientation $\alpha =24^{\circ }$, which is unseen during the training of the DNN, to evaluate the identification performance for a more realistic application scenario. Figure 12a shows the corresponding damage identification results using the complete feature vectors. It can be seen that the variations in these results are significantly increased in comparison to the results using the experiment-based DNN predictions; see Figure 11. Furthermore, the smallest thickness ratio τ=0.5 is often falsely identified for all damage orientations. These effects might be caused by the differences between the numerical and experimental WDIC patterns. Besides that, the proposed damage identification method uses a single damage metric to identify two different damage properties. It has been found that changes in the damage orientation α more strongly affect the WDIC pattern than changes in the thickness ratio τ [49]. Thus, the identified damage orientation might be more accurate in comparison to the damage thickness ratio.

However, in Figure 12b, the damage identification results using the compressed feature vector based on the anglewise PCA are presented. It can be seen that the feature compression significantly reduces the variation for both damage properties. In particular, the number of false identifications with the lowest thickness ratio τ=0.5 is decreased. The feature compression using anglewise PCA seems to reduce the influences of the differences between the experimentally measured and the numerically predicted WDICs and, thus, improves the results. Therefore, it seems reasonable to incorporate the computationally very efficient anglewise PCA into the proposed technique due to the trend to improve damage identification accuracy. Nevertheless, the performance should be verified to avoid an unexpected deterioration.

### 4.3. Discussion

The proposed damage identification method is tested in different cases using an experiment-based and a simulation-based approach. For the purely data-driven technique, the experimental training data might be affected by small imperfections, i.e., small position and orientation errors of the damage. Furthermore, SLDV measurements tend to be noisy due to the small guided wave amplitudes. This noise can be significantly reduced by averaging and further data processing, e.g., bandpass filtering [49]. These steps minimize the measurement error, which is not exactly quantifiable due to the unknown groundtruth. Nevertheless, the remaining measurement error influences the WDIC patterns as described by Humer et al. [49] and depicted in Figure 6d and Figure 9a. The experiment-based DNN predictions do not perfectly replicate the noisy WDIC patterns, which is partly intentional to prevent overfitting, i.e., fluctuations in the WDIC patterns caused by the measurement noise are also learned by the DNN. Generally, the measurement error has only minor effects on the damage identification method, which is underpinned by the excellent experiment-based identification results (see Figure 11) and high prediction accuracies (see Table 4). Although even a relatively small number of damage scenarios is sufficient to create and train the DNN, it might often be difficult or even impossible to measure the required data in practical SHM applications. In general, this limitation does not apply for a simulation-based approach, where the training data can be generated using numerical simulation models. The calculation of these data is typically very fast and cost-effective in comparison to experimental measurements using elaborate measurement equipment, e.g., an SLDV. However, numerical models require certain simplifications of the real damage scenario, e.g., in this study, the bonding layer between the artificial damage and the plate is simulated by an ideal tie connection. Hence, the numerical and experimental WDIC patterns show certain differences [49]. These differences influence the accuracy of the damage identification significantly, whereas the statistical analysis still indicates reasonable mean values of the damage properties.

However, this paper presents an anglewise PCA for reducing the feature dimensionality. Using this technique enables us to compress the feature vector on average by more than 90% for both the simulation- and experiment-based approach. Such a compressed feature vector with reduced dimensions might be useful in terms of several aspects. For example, if the experimental measurements of the unknown damage are corrupted by errors, e.g., due to temperature variations or measurement noise, this feature compression might help to reduce their effects. Nevertheless, the neglected scores of the anglewise PCA decrease the amount of information contained in the compressed feature vector. Therefore, the performance of the proposed feature compression technique should be verified to avoid an unexpected deterioration. However, the simple damage metric utilized herein could be replaced by an advanced machine learning algorithm. The general complexity and modeling effort, e.g., the size of the training data set, computational time for training, etc., of such algorithms can be significantly reduced with lower feature vector dimensionality. Besides that, it seems possible to implement a machine learning model, which learns the WDICs from training data to directly predict the damage properties of a given compressed feature vector.

In this study, the WDICs of an enormous number of different damages are predicted by the DNNs in a relatively short time, i.e., ad hoc. The calculation times for 9191 different damage scenarios and 1000 random triplets of sensing angles using a conventional desktop computer (HP EliteDesk 800 G5; Intel(R) Core(TM) i7-9700 with 3000 MHz and 32 GB RAM [72]) are listed in Table 8. It can be seen that both DNNs predict the various damage cases below 10 s for a random combination of three sensing angles, which might justify the designation ad hoc. The computation time for the anglewise PCA utilized for feature compression is almost negligible in comparison to the ones for the DNN predictions, while the damage identification, i.e., the evaluation of the damage metric, can basically be performed in real time. However, it seems counterintuitive that the simulation-based DNN needs more time to calculate the WDIC predictions despite its simpler architecture (7 × 80) in comparison to the experiment-based DNN (7 × 160). This can be explained by the increased computational effort due to more complex swish activation function. The total time for damage identification using the proposed approach is below 10 s despite the selected high number of 9191 different damage scenarios. Here, a very fine resolution was chosen for the predictions as a limiting scenario. However, the calculation times (see Table 8) already seem reasonable for practical SHM applications directly on site. A course resolution may allow for reducing computational efforts and, with it, damage identification times. Generally, the interval sizes used for damage identification are design parameters for the SHM system, where the actual choice may depend on several practical considerations.

However, the presented damage identification method assumes in each case a very challenging situation by using only three sensors and still performs mostly very well. It is assumed that including more sensors in the feature vector increases the contained damage information. This might further improve the reliability and accuracy of the presented damage identification method.

## 5. Conclusions

This paper presents a novel damage identification method based on experimental WDICs using ad hoc DNN predictions. These WDICs uniquely describe each damage while depending solely on its properties for a given host structure. Hence, they are utilized as highly sensitive damage features herein. The proposed damage identification methodology based on these WDICs is universally applicable and allows us to characterize certain properties of a damaged area. This is demonstrated using surface-bonded artificial damages that serve solely to prove the concept. Therefore, the experimental WDICs are extracted from SLDV measurements in laboratory experiments for twelve selected scenarios. This set of WDIC data is used to train a DNN. It is demonstrated that this fully trained DNN can precisely replicate the provided training data even for noisy and imperfect experimental data. Furthermore, the smart DNN interpolations enable predictions for exactly the requested sensing angles within a short time, i.e., ad hoc, for damages with arbitrary damage properties and frequency resolutions. The proposed damage identification method based on these ad hoc DNN predictions is tested in a challenging scenario using experimental data from only three sensors with random orientation. Additionally, a feature compression approach based on anglewise PCA is proposed to substantially reduce the feature dimensionality. The damage identification results using experiment-based DNN predictions are highly accurate even for damage unseen during offline training. In addition, a second DNN trained on simulated FE results is employed to predict the experimental WDICs. In this simulation-based approach, a decreasing identification performance is observed due to differences between the numerical and experimental WDIC patterns stemming from simplifications and noise. Applying the anglewise PCA for feature compression tends to reduce variations in the identified damage characteristics in this case. Overall, the results presented seem promising and highlight the potential of the proposed damage identification methodology for practical SHM applications.

In future research, the presented damage identification method could be applied to different structural damages, e.g., fatigue cracks in metals or delamination in composites. The phase information of the WDICs can be incorporated in the presented method to further increase the robustness and accuracy of the damage identification. The differences between the numerical and experimental WDICs could be addressed by using model-updating for creating a more accurate FE model. Finally, a transfer learning approach seems promising to improve the prediction accuracy of the already-trained simulation-based DNN with reasonable experimental efforts.

## Figures and Tables

**Table 1:** Damage properties from [50].

Damage No.	1	2	3	4	5	6	7	8	9	10	11	12
Orientation	0°	15°	30°	45°	0°	15°	30°	45°	0°	15°	30°	45°
Thickness Ratio	0.5				0.75				1			

**Table 2:** Material properties [50].

Parameter	Aluminum	Steel
Young’s modulus	70GPa	210GPa
Poisson’s ratio	0.33	0.3
Mass density	$2700\;{\mathrm{kg}/m}^{3}$	$7800\;{\mathrm{kg}/m}^{3}$

**Table 3:** Parameters for simulations and experiments with common dimensionless parameters.

Parameter	Simulation	Experiment
Damage size *D*	10mm	12.5mm
Plate thickness *h*	1.6mm	2mm
Damage thickness *t*	$\left\{0.8,1.2,1.6\right\}\;\mathrm{mm}$	$\left\{1,1.5,2\right\}\;\mathrm{mm}$
Wavelength $\lambda_{A_{0}}$	$\left\{10.68\ldots 6.75\right\}\;\mathrm{mm}$	$\left\{13.35\ldots 8.44\right\}\;\mathrm{mm}$
Frequency *f*	{120,122,…260}kHz	{96,97,…208}kHz
Dimensionless damage size-to-thickness ratio $\delta =\frac{D}{t}$	6.25	
Dimensionless damage-to-plate thickness ratio $\tau =\frac{t}{h}$	$\left\{0.5,0.75,1\right\}$	
Dimensionless damage size-to-wavelength ratio $\Lambda =\frac{D}{\lambda_{A_{0}}}$	$\left\{0.936\ldots 0.675\right\}$	
Characteristic product of frequency and half plate thickness $f\frac{h}{2}$	{96,97,…208}kHz-mm	

**Table 4:** Different measures of the prediction error for selected models, evaluated on the training data (12 damage scenarios; see Table 1), over all training data sets and an off-grid scenario ($\alpha =24^{\circ }$ and τ=0.625) unseen during training.

		7×160 with L-BFGS			7×160			7×190			8×190		
α	τ	**MAE**	**RMSE**	$R^{2}$	**MAE**	**RMSE**	$R^{2}$	**MAE**	**RMSE**	$R^{2}$	**MAE**	**RMSE**	$R^{2}$
$0^{\circ }$	0.5	0.0222	0.0330	0.9932	0.0488	0.0759	0.9639	0.0527	0.1027	0.9339	0.0667	0.1093	0.9251
$15^{\circ }$		0.0194	0.0268	0.9956	0.0466	0.0637	0.9753	0.0446	0.0654	0.9739	0.0531	0.0741	0.9665
$30^{\circ }$		0.0185	0.0264	0.9946	0.0435	0.0589	0.9732	0.0400	0.0538	0.9776	0.0409	0.0554	0.9763
$45^{\circ }$		0.0226	0.0344	0.9882	0.0471	0.0639	0.9594	0.0445	0.0616	0.9622	0.0433	0.0607	0.9633
$0^{\circ }$	0.75	0.0191	0.0271	0.9979	0.0434	0.0615	0.9893	0.0469	0.0664	0.9875	0.0569	0.0860	0.9790
$15^{\circ }$		0.0180	0.0248	0.9980	0.0439	0.0628	0.9869	0.0380	0.0542	0.9903	0.0474	0.0654	0.9858
$30^{\circ }$		0.0172	0.0237	0.9979	0.0431	0.0609	0.9864	0.0352	0.0470	0.9919	0.0423	0.0576	0.9878
$45^{\circ }$		0.0188	0.0269	0.9966	0.0439	0.0600	0.9831	0.0377	0.0504	0.9881	0.0409	0.0565	0.9850
$0^{\circ }$	1	0.0195	0.0283	0.9984	0.0448	0.0614	0.9924	0.0372	0.0502	0.9949	0.0445	0.0636	0.9918
$15^{\circ }$		0.0185	0.0268	0.9986	0.0434	0.0580	0.9935	0.0388	0.0521	0.9948	0.0438	0.0593	0.9932
$30^{\circ }$		0.0175	0.0253	0.9987	0.0408	0.0544	0.9938	0.0382	0.0509	0.9945	0.0421	0.0558	0.9934
$45^{\circ }$		0.0173	0.0242	0.9984	0.0414	0.0592	0.9902	0.0433	0.0684	0.9869	0.0469	0.0631	0.9889
all above		0.0191	0.0275	0.9978	0.0442	0.0619	0.9889	0.0414	0.0620	0.9889	0.0474	0.0689	0.9862
$24^{\circ }$	0.625	0.0205	0.0312	0.9964	0.0522	0.1078	0.9571	0.0409	0.0643	0.9847	0.0472	0.0741	0.9797

**Table 5:** Identified hyperparameters for DNN modeling.

Name	Experiment	Simulation
Optimizer	Adam + L-BFGS	
Mini-batch size	64 Samples	256 samples
Activation function	ReLU	Swish
Architecture	7×160	7×80
Number of parameters	155,521	39,361

**Table 6:** The reduced dimensions of the scores based on anglewise PCA ($r_{\mathrm{req}}=0.1$) from the original 113 experiment- and simulation-based features of a single sensing angle.

	Reduced Dimensions	3	4	5	6	7	8	9	10	11	12	13	14	15
Number of Directions	Experiment	1	12	24	27	19	23	20	11	4	3	1	0	0
	Simulation	0	0	3	12	12	20	29	20	15	18	12	1	3

**Table 7:** Damage identification results: mean value and standard deviation for damage properties τ and α from 10,000 random sensing angle combinations.

		Experiment-Based Predictions				Simulation-Based Predictions			
		**Full**		**Compressed**		**Full**		**Compressed**	
		**Feature Vector**		**Feature Vector**		**Feature Vector**		**Feature Vector**	
τ	α	$\bar{\tau }$	$\bar{\alpha }$	$\bar{\tau }$	$\bar{\alpha }$	$\bar{\tau }$	$\bar{\alpha }$	$\bar{\tau }$	$\bar{\alpha }$
		$s_{\tau }$	$s_{\alpha }$	$s_{\tau }$	$s_{\alpha }$	$s_{\tau }$	$s_{\alpha }$	$s_{\tau }$	$s_{\alpha }$
	[°]		[°]		[°]		[°]		[°]
0.5	0	0.5008	0.0114	0.5015	0.0269	0.5264	2.1491	0.5243	2.2434
		0.0020	0.0758	0.0035	0.1681	0.0583	6.3061	0.0577	6.0240
	15	0.5003	14.9996	0.5006	15.0015	0.5475	16.0671	0.5510	16.6346
		0.0012	0.1063	0.0019	0.1403	0.0793	8.7144	0.0793	7.6980
	30	0.5002	30.0016	0.5004	30.0043	0.5307	29.2781	0.5408	30.0807
		0.0010	0.0726	0.0015	0.1482	0.0492	10.3245	0.0538	9.1352
	45	0.5004	44.9734	0.5006	44.9525	0.5211	38.9824	0.5247	39.6663
		0.0015	0.1184	0.0019	0.1675	0.0514	11.7565	0.0538	10.0924
0.75	0	0.7500	0.0029	0.7503	0.0087	0.7088	3.2464	0.7049	3.6717
		0.0021	0.0376	0.0037	0.0680	0.1221	6.5041	0.1193	7.2355
	15	0.7499	15.0013	0.7496	15.0072	0.6792	16.2795	0.7085	16.5394
		0.0019	0.0771	0.0030	0.1251	0.1393	8.2579	0.1344	7.5834
	30	0.7501	30.0112	0.7502	30.0120	0.6967	30.4075	0.7194	31.0561
		0.0018	0.1016	0.0027	0.1532	0.1408	10.6367	0.1321	9.1931
	45	0.7500	44.9911	0.7501	44.9722	0.6905	37.7519	0.7190	38.8593
		0.0018	0.0665	0.0030	0.1155	0.1326	12.5055	0.1223	10.9120
1	0	0.9996	0.0060	0.9991	0.0111	0.8749	3.9274	0.9081	2.8385
		0.0016	0.0544	0.0024	0.0742	0.1762	9.1318	0.1361	7.3066
	15	0.9996	14.9956	0.9990	14.9841	0.8535	16.7008	0.8808	16.2203
		0.0014	0.0878	0.0026	0.1416	0.1854	8.5646	0.1597	7.7407
	30	0.9996	29.9980	0.9989	30.0012	0.8664	31.6611	0.8776	30.5236
		0.0014	0.0792	0.0024	0.1398	0.1676	8.1846	0.1572	8.6023
	45	0.9998	44.9881	0.9994	44.9700	0.9028	40.1049	0.9121	40.1234
		0.0014	0.0793	0.0018	0.1261	0.1699	10.0021	0.1560	9.1273
0.625	24	0.6251	23.9962	0.6252	23.9763	0.6545	22.7689	0.6839	23.0496
		0.0019	0.0999	0.0032	0.1864	0.1331	9.5437	0.1283	8.4294

**Table 8:** Calculation time for DNN prediction, anglewise PCA and damage identification for 9191 predicted damage scenarios and 1000 random combinations of three sensing angles using a conventional desktop computer.

Task	Mean Time [s]	Standard Deviation [s]
Experiment-based DNN prediction	6.5	0.2
Simulation-based DNN prediction	8.2	0.5
Anglewise PCA	0.2	0.1
Damage identification	≪0.1	≪0.1

## Data Availability

Data set available on request from the authors.

## Abbreviations

The following abbreviations are used in this manuscript:

CAFA	Combined analytical finite element method approach
DNN	Deep neural network
FE	Finite element
L-BFGS	Limited-memory Broyden–Fletcher–Goldfarb–Shanno
MSE	Mean square error
PC	Principal component
PCA	Principal component analysis
PWAS	Piezoelectric wafer active sensor
ReLU	Rectified linear unit
RMSE	Root mean square error
SGD	Stochastic gradient descent
SHM	Structural health monitoring
SLDV	Scanning laser Doppler vibrometer
WDIC	Wave damage interaction coefficient
